# Role of Lipid-Lowering and Anti-Inflammatory Therapies on Plaque Stabilization

**DOI:** 10.3390/jcm13113096

**Published:** 2024-05-25

**Authors:** Krzysztof L. Bryniarski, Wijnand den Dekker, Jacek Legutko, Pawel Gasior, Jeroen Tahon, Roberto Diletti, Jeroen M. Wilschut, Rutger-Jan Nuis, Joost Daemen, Pawel Kleczynski, Nicolas M. Van Mieghem, Ik-Kyung Jang

**Affiliations:** 1 Department of interventional Cardiology, Thoraxcenter, Cardiovascular Institute, Erasmus University Medical Center, 3000 CA Rotterdam, The Netherlands; 2 Department of Interventional Cardiology, Jagiellonian University Medical College, Institute of Cardiology, St. John Paul II Hospital, 31-202 Krakow, Poland; 3 Division of Cardiology and Structural Heart Diseases, Medical University of Silesia in Katowice, 40-055 Katowice, Poland; 4 Department of Interventional Cardiology, Imelda Hospital, 2820 Bonheiden, Belgium; 5 Cardiology Division, Massachusetts General Hospital, Harvard Medical School, Boston, MA 02115, USA

**Keywords:** coronary artery disease, lipid-lowering therapy, plaque vulnerability

## Abstract

Atherosclerosis is the predominant underlying etiopathology of coronary artery disease. Changes in plaque phenotype from stable to high risk may spur future major adverse cardiac events (MACE). Different pharmacological therapies have been implemented to mitigate this risk. Over the last two decades, intravascular imaging modalities have emerged in clinical studies to clarify how these therapies may affect the composition and burden of coronary plaques. Lipid-lowering agents, such as statins, ezetimibe, and proprotein convertase subtilisin/kexin type 9 inhibitors, were shown not only to reduce low-density lipoprotein levels and MACE but also to directly affect features of coronary plaque vulnerability. Studies have demonstrated that lipid-lowering therapy reduces the percentage of atheroma volume and number of macrophages and increases fibrous cap thickness. Future studies should answer the question of whether pharmacological plaque stabilization may be sufficient to mitigate the risk of MACE for selected groups of patients with atherosclerotic coronary disease.

## 1. Introduction

Ischemic heart disease (IHD) is the leading cause of death and disability worldwide [1]. Atherosclerosis is the principle underlying the etiopathology of coronary artery disease (CAD) and IHD. The introduction of intravascular imaging into clinical practice allowed for the in vivo assessment of CAD, including the mechanism responsible for coronary atherosclerotic plaque progression and destabilization. By using intravascular modalities, we are able to detect atherosclerotic plaques, which are at high risk of rapid progression, leading to subsequent coronary events. However, despite the development of modalities used to visualize in vivo coronary plaques during angiography, as well as major advances in both interventional and pharmacological treatment of CAD, treating non-obstructive, non-culprit vulnerable plaques remains a challenge. Currently, two different treatment pathways have been proposed: (1) pharmacological therapy and (2) mechanical stabilization of the plaques. Whereas medical therapy is well established and supported by clinical guidelines, mechanical stabilization of non-culprit, hemodynamically not significant plaques is still being evaluated in clinical trials. In this review, we will discuss pharmacological treatment options for atherosclerotic plaques, describe how this treatment may affect plaque morphology, and summarize clinical trials assessing plaque regression.

## 2. Treatment of Atherosclerotic Plaques

### 2.1. Diagnostic Modalities for Vulnerable Plaque Detection

Hypertension, diabetes, smoking, stress, and pollution may promote plaque formation [2,3]. The initial steps of atherogenesis include endothelial dysfunction and abnormal lipid metabolism, which are caused by inflammation. Pathological intimal thickening (PIT) may transform into an atheroma with the appearance of a lipid pool. Inflammatory processes catalyze the transformation into a thin cap fibroatheroma (TCFA). TCFA consists of a necrotic core and a thin fibrous cap infiltrated by macrophages and lymphocytes and features type I collagen with few or no smooth muscle cells; this is the hallmark of a vulnerable plaque [4]. Macrophages derived from migrated monocytes or smooth muscle cells phagocytose oxidized low-density lipoprotein (LDL) to become foamy cells and to form fatty streaks and lipid cores [5]. TCFA has been initially found most frequently in pathology specimens obtained from victims of fatal ACS. The introduction of intravascular imaging allowed for the determination of an in vivo link between features of plaque vulnerability and adverse cardiac events (Table 1). Intravascular ultrasound (IVUS) was the first modality to be frequently used in daily clinical practice. IVUS studies linked positive remodeling and greater plaque burden to unstable CAD [6]. However, because of its low resolution, IVUS cannot detect a fibrous cap. Thus, the positive predictive value of IVUS for detecting TCFA is very low [7]. To facilitate the interpretation of the images of different tissue components, autoregressive spectral analysis was added to IVUS [8]. Virtual histology intravascular ultrasound (VH-IVUS) was pivotal for understanding the features of plaque vulnerability. Prospective studies such as the PROSPECT, VIVA, and ATHEROREMO-IVUS revealed that several properties of coronary plaque, including a large plaque burden, a TCFA, or a defined minimal lumen area (≤4.0 mm^2^ in the PROSPECT study), were independent predictors of future major adverse cardiac events (MACE) during long-term follow-up [9,10,11].

The need for even more detailed evaluations of coronary lesions prompted the introduction of a new modality, near-infrared spectroscopy (NIRS). NIRS provides information on lipid content in the arterial wall and thus shows the presence of lipid-rich plaques by providing the lipid core burden index (LCBI) [12]. Numerous studies have demonstrated that a larger LCBI_4mm_ is an independent predictor of future MACE [13,14].

Currently, high-resolution optical coherence tomography (OCT) seems to be the gold standard for identifying the traits of vulnerable plaques in vivo [15]. Lipid-rich plaques and TCFA, as defined by a cap thickness < 65 µm, are key features of plaque vulnerability detected using OCT [16,17]. What is more, in the CLIMA study, macrophages, which are a marker of inflammation, were associated with a greater incidence of MACE [18]. 

Coronary CT angiography (CCTA) is widely used in patients with suspected CAD. This modality may also be useful in the detection of vulnerable plaques [19]. Observational studies have shown that plaque features detected by CCTA, such as spotty calcification, positive remodeling, low plaque attenuation, and the napkin sign, may be associated with an increased risk of adverse cardiac events [20,21]. Similarly, the PROMISE trial showed that low attenuation, positive remodeling, and the napkin-ring sign were linked with an increased risk of cardiovascular disease during follow-up [22]. There appears to be a good correlation between CCTA and OCT in terms of plaque characterization [23,24].

### 2.2. Approach to Plaque Stabilization

Patients with plaque progression have a noticeably greater risk of future cardiac coronary events than those with stable plaques [2,25]. The prevention of plaque progression in the early stages is a feasible method of reducing cardiovascular risk in the future. Currently, two different treatment pathways have been proposed: (1) pharmacological therapy and (2) mechanical stabilization of the plaques. 

Pharmacological therapy is used for the stabilization of both significant and nonsignificant lesions in daily clinical practice worldwide. Currently, pharmacological therapy is based on medications that reduce cholesterol deposition and inflammation. Reduced cholesterol deposition is achieved with lipid-lowering agents, such as statins (HMG-CoA reductase inhibitors), ezetimibe (which inhibit intestinal and biliary cholesterol absorption), and proprotein convertase subtilisin/kexin type 9 inhibitors (which increase hepatocyte uptake of LDL-C). Use of those medications is supported by European Society of Cardiology (ESC) guidelines [19,26]. Reduction of inflammation in the cardiovascular bed is mainly achieved with colchicine (microtubule formation inhibitor) and eicosapentaenoic acid (modulator dampening inflammatory response). Numerous studies presented in this review demonstrated the effect of pharmacological therapy on plaque stabilization. Of note, it should be emphasized that other medications that are beyond the scope of this review are also used in patients with CAD in order to reduce the risk of MACE. Those medications include inter alia beta-blockers, angiotensin-converting enzyme inhibitors, antiplatelet agents, and oral anticoagulation [26,27]. Importantly, several studies demonstrated that an initial conservative strategy in terms of MACE may be comparable to an initial invasive strategy. In the COURAGE trial, 2287 patients with stable angina were randomized into PCI with an optimal medical therapy group or to optimal medical therapy alone [28]. After 4.6 years of follow-up, there were no differences in the composite of death, myocardial infarction, and stroke between the two groups. However, there was a significant difference in rates of revascularization between PCI and optimal medical therapy alone arms. Comparable results were presented in the ISCHEMIA trial where 5179 patients were included and followed up for 3.2 years [29]

Mechanical stabilization is used predominantly for culprit lesions in patients with ACS as well as for obstructive non-culprit lesions in patients with ACS and chronic coronary syndrome (CCS). Further research continues to evaluate the mechanical stabilization of non-culprit, hemodynamically non-significant plaques [30,31]. 

## 3. Statins

Statins reduce the synthesis of cholesterol in the liver and promote LDL receptor (LDLR) expression at the surface of hepatocytes, which subsequently results in increased uptake of LDL from the blood and decreased plasma concentrations of LDL- and other ApoB-containing lipoproteins, including triglyceride-rich particles [32]. High-intensity statin treatment may reduce LDL levels by up to 50%. Statins have pleiotropic effects that include the inhibition of oxidation-sensitive inflammatory pathways, the modulation of leukocyte–endothelial cell interactions, and the reduction of inflammatory cytokine levels [33,34,35]. Statin therapy clearly reduces the risk of MACEs in patients with CVD [27,36,37].

**Figure 1:**
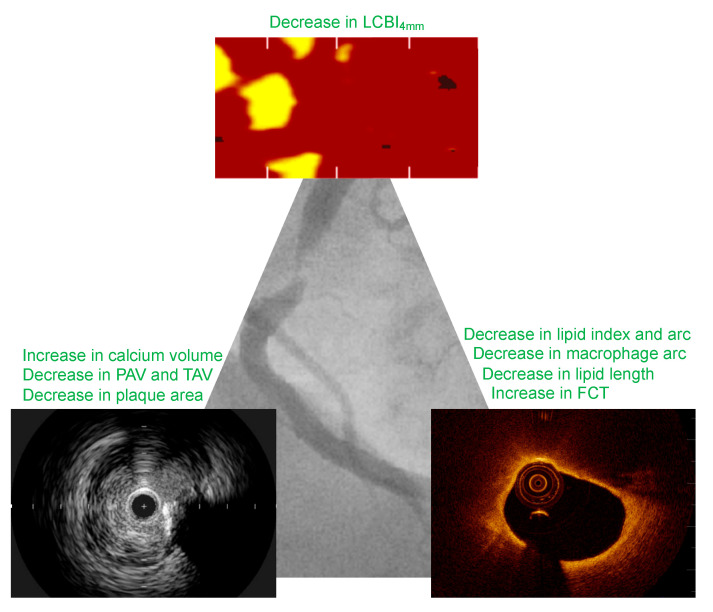
Major effects of lipid-lowering therapy on coronary plaque morphology. FCT, fibrous cap thickness.

In 1997, the first randomized trial comparing the progression of atherosclerotic plaques between patients with and without statin treatment was reported (Table 2) [38]. Only 36 patients were enrolled in the study, but there was a significant reduction in atheroma volume on IVUS after 36 months of initial therapy (−7% vs. +41%; *p* < 00.1). In the ASTEROID trial, an open-label blinded endpoint multicenter trial, 507 statin-naive patients were recruited [39]. Serial IVUS examinations were performed for 349 patients during the 2-year follow-up. All patients received high-intensity statin therapy (40 mg rosuvastatin) during the trial period. The authors found a significant change in percent atheroma volume (PAV) compared to the index procedure (0.79%; 95.5% CI, −1.21% to −0.53%; *p* < 0.001) and a significant change in total atheroma volume (TAV) (−12.5 mm^3^; 95% CI, −15.1 to −10.5 mm^3^, *p* < 0.001) (Figure 1). Similar results were achieved in the IVUS IBIS-4 trial [40]. This study analyzed the response of non-culprit lesions to treatment with 40 mg rosuvastatin in patients after ST-elevation myocardial infarction (STEMI). The PAV reduction was −0.9% (95% CI; −1.56 to −0.025, *p* = 0.007); however, both the percentage of necrotic core and the number of TCFAs remained the same. Furthermore, in a Japanese cohort, Takayama et al. reported a 5.1% change in plaque volume in patients receiving 2.5 mg of rosuvastatin daily (the dose of rosuvastatin could increase after 4 weeks to a maximum daily dose of 20 mg) [41].

Not only is starting therapy with statins crucial for CAD patients, but the dose and type of statins are also important. A trial including more than 1000 patients who had serial IVUS measurements performed at baseline and after 104 weeks demonstrated that the TAV was lower with rosuvastatin therapy (40 mg) than with atorvastatin therapy (80 mg) (−6.39 mm^3^; 95% CI, −7.52 to −5.12 vs. −4.42 mm^2^; 95% CI, −5.98 to −3.26; *p* = 0.01) [42,56]. PAV was not different between the two groups (0.99% vs. 1.22%; *p* = 0.17), and both agents induced plaque regression in most patients (63.2% vs. 68.5%, *p* = 0.02). However, these results demonstrated that plaque progression occurred in approximately one-third of the patients, regardless of the type or dose of statin. Further analysis of these results demonstrated that high blood pressure, diabetes, increased levels of apoB, and decreased levels of HDL-C were associated with ongoing plaque progression despite adequate statin therapy [57]. In another study comparing pravastatin (40 mg) with atorvastatin (80 mg), the latter significantly reduced the PAV and TAV [58]. Notably, coronary atherosclerosis progressed in the pravastatin group but not in the atorvastatin group.

Pooled analysis of angiographic lipid-lowering trials before the era of intravascular imaging showed that patients had a 22% to 34% reduction in cardiac events [59]. In contrast, small angiographic regression of atherosclerotic lesions in these trials was not paired with event reduction; therefore, it was unlikely that angiographic regression itself caused such a large clinical benefit. Indeed, as demonstrated by a large number of studies, statins not only cause plaque regression but also modify features of plaque vulnerability. Authors in the previously described SATURN trial using VH-IVUS analysis observed that high-intensity statin therapy was associated with small reductions in fibrous and fibrofatty tissue, with an increase in dense calcium and without influence on necrotic core volume [56,60]. These results are consistent with a meta-analysis of nine statin treatment studies that demonstrated a reduction in fibrous plaque volume as well as an increase in dense calcium volume [61]. Notably, in this meta-analysis, the authors failed to observe changes in fibro-fatty and necrotic core volumes.

Both OCT and NIRS provided further insight into changes in vulnerable plaque composition in patients treated with statins. Katoka et al. showed that the coronary plaques of patients receiving statin therapy have a smaller lipid arc and greater fibrous cap thickness [48]. Several OCT clinical trials and meta-analyses confirmed that high-intensity statin treatment is associated with greater fibrous cap thickness and a reduced lipid arc [4,49,62]. Furthermore, Chia et al. reported that patients on established statin therapy had fewer plaque ruptures than did statin-naive patients (8 vs. 33%; *p* = 0.03) [63]. In the IBIS-4 trial, 103 patients with STEMI underwent imaging with IVUS and OCT of two non-infarct coronary arteries [51]. All of the patients were treated with high-dose rosuvastatin. At the 13-month median follow-up, the authors found that fibrous cap thickness increased, whereas both the macrophage line arc and lipid arc decreased. Moreover, 9 out of 13 TCFAs from baseline regressed to non-TCFAs, and only 2 out of 178 non-TCFAs progressed to a TCFA. Finally, Nishiguchi et al. analyzed 53 patients who were randomized to either the early or late pitavastatin group (4 mg of pitavastatin in both groups; one group started receiving statins at baseline, and the second group started receiving statins 3 weeks after the baseline procedure). OCT was performed at baseline and 3 and 36 weeks after the baseline procedure. Between baseline and the 3-week follow-up, fibrous cap thickness increased in the early statin group and decreased in the late statin group (8.3% vs. −5.8% increase; *p* < 0.001).

Notably, a recent OCT study demonstrated that predictors of favorable vascular response to statin therapy included a large thin-cap area, a high macrophage index, and a layered plaque phenotype [64]. In conclusion, OCT studies demonstrated that statin therapy may not only increase fibrous cap thickness and decrease the lipid arc but also cause a reduction of macrophages.

The YELLOW trial assessed the impact of short-term intensive statin therapy (40 mg rosuvastatin) on intracoronary plaque lipid content detected using NIRS [53]. Patients were randomized to either high-dose statin treatment or standard-of-care statin treatment. After 7 weeks, patients in the intensive statin group had a greater reduction in the LCBI_4mm_ than did those in the standard therapy group (median reduction −149.1 vs. 2.4; *p* = 0.01). Notably, after this short follow-up, only changes in the LCBI_4mm_ were observed without any changes in plaque burden. Nevertheless, this study showed that even short-term statin therapy may decrease plaque vulnerability.

### 3.1. Ezetimibe

Ezetimibe inhibits intestinal uptake of dietary and biliary cholesterol at the level of the brush border of the intestine without affecting the absorption of fat-soluble nutrients [32]. Ezetimibe has a different metabolic pathway than statins—it reduces cholesterol uptake and delivery to the liver, which subsequently upregulates LDLR expression and hence increases the clearance of LDL from the blood. Ezetimibe decreases LDL levels by an additional 21–27% when added to statin therapy [65]. Importantly, combining ezetimibe with statins reduces MACE rates [19,27,66].

Similarly to statin trials, intravascular ultrasound studies assessed plaque modification by adding ezetimibe to statin therapy. In the PRECISE-IVUS trial, 202 patients were randomized to receive either atorvastatin therapy or atorvastatin with 10 mg ezetimibe therapy. Dual therapy significantly decreases the PAV (−1.4% vs. −0.3%; *p* = 0.001) at the 1-year follow-up [45]. Moreover, a significantly greater number of patients on dual therapy experienced coronary plaque regression (78% vs. 58%; *p* = 0.004). Additionally, in the HEAVEN VH-IVUS trial, statin and ezetimibe as opposed to statin monotherapy decreased the PAV (−0.4% vs. +1.4%; *p* = 0.014) compared with statin monotherapy; however, there were no significant changes in plaque composition [43].

Conversely, the ZEUS trial did not observe significant changes in the PAV between patients receiving statin and ezetimibe therapy and those receiving statin therapy alone (−12.5% vs. −7.6%; *p* = 0.06) [44]. However, only 95 patients were recruited for the study, and follow-up IVUS was performed after 6 months, which could have affected the results. A small OCT study revealed increased fibrous cap thickness and a decreased lipid angle with fluvastatin and ezetimibe compared with fluvastatin alone [67].

### 3.2. Proprotein Convertase Subtilisin/Kexin Type 9 (PCSK9) Inhibitors

PCKS9 inhibitors reduce plasma levels of PCKS9, which substantially lowers LDL levels [32]. These drugs reduce LDL levels by 60% alone or up to 85% when combined with statins and ezetimibe and reduce the MACE rate by up to 15% [32,68].

The largest trial assessing coronary plaque modification when using PCSK9 inhibitors was the GLAGOV trial [47]. Altogether, 968 patients were randomly allocated to evolocumab and statins or placebo and statins. Angiographic follow-up with IVUS was performed at 76 weeks (331 patients were eligible for IVUS measurements). PAV increased by 0.05% in the placebo group and decreased by 0.95% in the evolocumab group (*p* < 0.01). Evolocumab induced plaque regression in 64.3% of patients compared to 47.3% of patients in the placebo group (*p* < 0.01). Notably, the authors observed a direct relationship between lowered LDL levels and plaque regression even at LDL levels as low as 20 mg/dL. This indicates that they did not find a threshold for lipid-lowering therapy, at which point, further LDL lowering was not beneficial. Of the 968 patients in the GLAGOV trial, 331 had VH-IVUS measurements [69]. Although the authors did not observe differences in plaque composition between the two treatment groups, they observed an inverse correlation between changes in LDL cholesterol levels and plaque calcification (r = 0–15; *p* < 0.001). The same observation was made in statin studies where statin treatment was associated with an increase in plaque calcification [34,70,71]. Thus, it is suggested that plaque calcification may be due to lipid-lowering effects and not due to the pleiotropic properties of statins.

Whereas patients included in the GLAGOV trial had stable angina, the PACMAN-AMI trial included patients with myocardial infarction (MI) [54]. Among the 300 patients included in the study, those who were randomized to the alirocumab with optimal medical therapy (OMT) group had greater PAV reduction than patients in the placebo with OMT group (−2.13% vs. −0.92%; *p* < 0.001) at 52 weeks of follow-up. Plaque regression is typically greater in ACS patients than in stable patients, which is also consistent with greater plaque burden at baseline in patients who present with MI. The PACMAN-AMI study used three modalities to evaluate coronary plaques. The change in fibrous cap thickness was greater in the alirocumab group (62.67 μm vs. 33.19 μm; *p* = 0.001), and there was a greater reduction in the mean angular extension of macrophages in alirocumab-treated patients (−25.98 vs. −15.95; *p* < 0.001). However, the study failed to show differences in the LCBI_4mm_ (−79.42 vs. 37.60; *p* = 0.006). OCT was also used in a study by Nicholls et al., in which patients who underwent NSTEMI were randomized to either the evolocumab group or the placebo group [55]. Those who were treated with evolocumab had a greater increase in the minimum fibrous cap thickness, a greater decrease in the maximum lipid arc, and a greater decrease in the macrophage index.

Recently, inclisiran was introduced for lipid-lowering therapy and was shown to reduce LDL levels by 50% [72]. The effect of long-term follow-up on MACE reduction in a large population is being assessed. Inclisiran is a small interfering RNA that prevents PCSK9 production [34].

### 3.3. Omega-3 Fatty Acids

The mechanism of action of eicosapentaenoic acid (EPA) is poorly understood; however, it may be partially related to its ability to interact with peroxisome proliferator-activated receptors and decrease the secretion of ApoB [32]. In the CHERRY trial, Watanabe et al. recruited 193 patients and divided them into pitavastatin or pitavastatin with EPA (1800 mg daily) groups [46]. After 6–8 months of follow-up, EPA had a greater positive effect on the TAV reduction, as assessed by IVUS. Similar results were demonstrated in the study by Niki et al., with statistically significant reductions in lipid volume as well as an increase in fibrous plaque volume during follow-up in patients with EPA treatment [73]. In an OCT study, patients administered rosuvastatin with EPA had a greater reduction in the lipid index and macrophages than patients administered rosuvastatin alone [52]. However, in this study, patients received different doses of statins. Those who were administered EPA also received 10 mg of rosuvastatin, whereas those in the control group received only 2.5 mg of rosuvastatin. Another OCT study showed that EPA or EPA with docosahexaenoic acid (DHA) therapy in addition to strong statin therapy did not significantly increase FCT in non-culprit plaques compared with strong statin therapy alone [74]. In this study, a relatively small number of the 130 patients were divided into three groups: statin; statin with high-dose EPA; and statin with EPA and DHA. In a recent study, icosapent ethyl reduced MACE risk regardless of lipoprotein levels [75]. Finally, in the CCTA trial group, patients receiving 4 g of icosapent ethyl daily in addition to OMT had greater plaque regression than patients receiving OMT only [76].

## 4. Colchicine

Colchicine is an old medication, and its anti-inflammatory properties were recognized even in the New Kingdom Era in Egypt (circa 1500 years BC) [77]. It inhibits microtubule formation and the polymerization of tubulin and hence suppresses the inflammatory response [2]. In the vascular bed, colchicine reduces the migration, adhesion, and activation of neutrophils in inflamed endothelium, suppresses the assembly and activation of NLRP3, and reduces inflammatory cytokines that are connected with the development of vulnerable plaques [78,79]. Colchicine reduces MACE rates in patients with CAD [80]. In the COLCOT trial, 4745 ACS patients were randomized to receive low-dose colchicine (0.5 mg daily; 2366 patients) or placebo (2379 patients). After a median follow-up of 22.6 months, the primary endpoint (death from cardiovascular causes, resuscitated cardiac arrest, MI, stroke, and urgent revascularization for angina leading to PCI) occurred in 5.5% of patients in the colchicine group vs. 7.1% of patients in the placebo group (*p* = 0.02). Notably, gastrointestinal events were less common than expected. The ESC guidelines recommend colchicine for secondary prevention of cardiovascular disease (class IIb) [27]. In the CCTA study, colchicine resulted in a significant decrease in low attenuation plaque volume as well as in high-sensitivity CRP levels [81]. No difference in total atheroma volume reduction was observed (42.3 mm^3^ vs. 26.4 mm^3^; *p* = 0.28). In the ongoing COCOMO-ACS study, 64 patients with or without colchicine therapy after MI will be evaluated with OCT at baseline and after 18 months [82]. Nevertheless, further studies are warranted to assess the influence of colchicine on coronary plaque progression.

### 4.1. Other Medications

Inhibition of IL-1 may be achieved by using anakinra receptor antagonists [83]. Anakinra was shown to decrease the area under the curve (AUC) of CRP levels and decrease the death rate and new onset or worsening of heart failure in patients after STEMI [84]. Tocilizumab, an anti-IL-6R antibody, attenuated the inflammatory response and PCI-related troponin release in NSTEMI patients [85]. However, tocilizumab causes an increase in triglycerides. Currently, ziltivekimab is assessed in the ZEUS trial [86]. The human monoclonal antibody canakinumab, which targets interleukin-1β, was evaluated in the CANTOS trial. The study involved 10,000 patients who were followed for 3 years [87]. The use of canakinumab at a dose of 150 mg every 3 months led to a 15% reduction in the rate of MACEs compared to that in the placebo group. Importantly, this effect was independent of decreasing lipid levels. The CANTOS trial demonstrated that inflammation inhibition may prevent atherosclerosis-related events in humans. Patients treated with canakinumab had a lower incidence of cancer, particularly a decrease in mortality from lung cancer. However, an increase in the infection rate was observed in patients who received canakinumab. Methotrexate is another drug that has been tested for its ability to reduce the incidence of CVD. However, a study involving low-dose methotrexate did not show a reduction in CRP levels or coronary events [88]. To our knowledge, up to date, there are no in vivo studies assessing changes in plaque morphology when using those agents. Interleukin 6 is secreted by macrophages, monocytes, fibroblasts, and endothelial cells. It has both pro-inflammatory and pro-thrombotic properties [3]. It may promote both atherosclerosis and plaque vulnerability. Similarly, interleukin-1β may play a role as one of the key steps in the inflammatory signaling process. In vitro evidence suggests that this cytokine may act as a regulatory protein in the atherosclerotic process [89]. The role of inflammatory pathways in atherosclerosis was described in detail by Soehnlein et al. and Dimitroglou et al. [3,90].

### 4.2. Mechanical Stabilization

ACS most commonly arises from vulnerable plaques [4]. Thus, the concept of preventive PCI of non-flow-limiting highly vulnerable plaques has emerged in recent years. Stone et al. in their study randomized 182 patients with angiographically non-obstructive lesions with plaque burden greater than 65% to either PCI (with bioresorbable vascular scaffold) with optimal medical therapy or optimal medical therapy alone [30]. After 25 months of follow-up, patients in the PCI group had a greater minimal lumen area. However, this study was not powered for clinical outcomes. Moreover, no other features of plaque vulnerability such as TCFA were assessed in this study. In the recently published PREVENT trial, 1606 patients with non-flow-limiting lesions (assessed with fractional flow reserve) and features of plaque vulnerability were randomly assigned to PCI or OMT alone [31]. In this trial, the assessment of features of plaque vulnerability included the use of IVUS, NIRS, and OCT. At 2 years follow-up, patients in the PCI group had a lower risk of MACE. Howbeit, in most of the patients IVUS was used, and thus vulnerable plaque was mostly recognized by high plaque burden and not other features such as TCFA or lipid plaque. Further studies evaluating mechanical stabilization in non-flow-limiting lesions are underway. 

## 5. Conclusions

Various studies have established the role of lipid-lowering therapies and anti-inflammatory therapies in mitigating plaque progression or even inducing plaque regression. Furthermore, increasing the intensity of statin therapy or combining different agents can achieve better results in terms of changes in plaque composition. New imaging modalities have allowed us to better understand in vivo changes in plaque morphology and how these changes may influence the MACE rate. In the near future, we will determine whether these modalities are more frequently used to assess patients’ coronary plaque vulnerability risk and hence tailor targeted therapy. Future studies should answer the question of whether plaque stabilization by medical treatment alone without invasive procedures may be feasible for selected groups of patients.

## Figures and Tables

**Table 1:** Definitions of most common features of plaque vulnerability.

	GS-IVUS	VH-IVUS	NIRS	OCT
Fibroatheroma/Lipid plaque	Can identify lipid plaque—so called “soft” plaque which is described as an area with low echogenicity in contrast to the reference adventitia.	VH-IVUS cannot directly identify fibroatheroma. Fibroatheroma is described as the presence of 10% confluent necrotic core with an overlying layer of fibrous tissue on 3 consecutive frames (1).	Shows probability of lipid as yellow pixels on chemogram and lipid core burden index (LCBI). LCBI can be calculated in any chosen segment as the proportion of yellow pixels to all pixels in the chosen area multiplied by 1000. LCBI_4mm_ refers to the value of LCBI in chosen 4 mm segment of the artery.	Can identify lipid plaque described as signal-poor regions with diffuse borders (lipid pool) and overlying signal-rich bands (fibrous caps), accompanied by high signal attenuation. Due to this limitation, it is frequently not possible to assess the diameter of the artery with lipid plaque.
TCFA	GS-IVUS does not have resolution high enough to visualize TCFA.	VH-IVUS cannot identify TCFA directly. TCFA is described as the presence of 10% confluent necrotic core in direct contact with the lumen on 3 consecutive frames (1).	NA	Lipid plaque with the minimum thickness of the fibrous cap less than 65 μm or 80 μm and with lipid occupying >90° in circumference.
Plaque burden	Percentage of the plaque area within the entire vessel wall	Percentage of the plaque area within the entire vessel wall	NA	NA
Macrophages	NA	NA	NA	Increased signal intensity within the plaque, accompanied by heterogeneous back shadows

GS-IVUS, greyscale intravascular ultrasound; NIRS, near-infrared spectroscopy; OCT, optical coherence tomography; TCFA, thin-cap fibroatheroma; VH-IVUS, virtual histology intravascular ultrasound. Adapted from Legutko et al. [4]. (1) Necrotic core on VH-IVUS is visible as red pixels, calcium is visible as white pixels.

**Table 2:** Studies assessing lipid-lowering therapy on changes in coronary plaque morphology.

Study/Publication Year	Study Size	Treatment	Follow-Up Time	Changes in Plaque Composition
IVUS, VH-IVUS				
Takagi et al. [38] 1997	36 patients	10 mg pravastatin + diet vs. diet alone	3 years	Mean change of lumen area: +10 vs. −9% (*p* < 0.001) Mean change of plaque area: −7 vs. +41% (*p* < 0.001)
Nissen et al. [39] ASTEROID Trial 2006	507 patients (349 included in follow-up) with stable and unstable ischemic chest pain	Rosuvastatin 40 mg	2 years	Values compared to baseline Median PAV decrease: −0.79% (*p* < 0.001) Median change of atheroma volume in 10 mm prespecified segment with greatest disease: −5.6 mm^3^ (*p* < 0.001) Median TAV change: −12.5 mm^3^ (*p* < 0.001) 63.6% of patients showed regression of the disease and 36.4% progression of the disease
Takayama et al. [41] COSMOS 2009	214 patients (126 included in follow-up)	Rosuvastatin 2.5 mg (could be increased after 4 weeks)	76 weeks	Values compared to baseline Mean PAV change: −5.1% (*p* < 0.001) Mean plaque area change: −21.9 mm^3^ (*p* < 0.001)
Nicholls et al. [42] SATURN 2011	1039 patients with both SA and ACS	Rosuvastatin 40 mg vs. atorvastatin 80 mg	24 months	Median PAV change: −1.22 vs. −0.99% (*p* = 0.17) Median TAV change: −6.39 vs. −4.42% (*p* = 0.01) Disease regression (based on TAV): 71.3 vs. 64.7% (*p* = 0.02)
Kovarnik et al. [43] HEAVEN 2012	89 patients with SA	Atorvastatin 80 mg + ezetimibe 10 mg vs. routine statin therapy (10 mg atorvastatin in statin naïve patients)	12 months	Mean PAV change: −0.4 vs. 1.4% (*p* = 0.014) Mean necrotic core change: 1.5 vs. 3.4% (*p* = 0.18) Mean calcification change: 1.0 vs. 2.6% (*p* = 0.18)
Nakajima et al. [44] ZEUS 2014	95 patients with ACS	Atorvastatin 20mg + ezetimibe 10 mg vs. atorvastatin 20 mg	24 weeks	Mean plaque volume change: −12.5 vs. −7.5% (*p* = 0.06) Mean vessel volume change: −7.4 vs. −2.0% (*p* = 0.04)
Raber et al. [40] IBIS-4 2015	103 patients with STEMI	Rosuvastatin 40 mg	13 months	Values compared to baseline Mean PAV change: −0.9% (*p* = 0.007) Mean TAV change: −13.4 mm^3^ (*p* = 0.006) Mean NC volume change: −0.05% (*p* = 0.926) Mean dense calcium change: 1.28% (*p* < 0.001) Number of TCFA: 124 vs. 116 (*p* = 0.15)
Tsujita et al. [45] PRECISE-IVUS 2015	202 patients with SA and ACS	Atorvastatin + 10 mg ezetimibe vs. atorvastatin	9–12 months	Median plaque volume change: −5.2 vs. −1.3% (*p* < 0.001) Median TAV change: −6.6 vs. −1.4% (*p* < 0.001) −10.2 vs. −1.3% (*p* < 0.001) in ACS group −5.0 vs. −1.5% (*p* = 0.008) in SA group
Watanabe et al. [46] CHERRY 2017	193 patients with SA and ACS	Pitavastatin 4 mg + EPA 1800 mg vs. pitavastatin 4 mg	6–8 months	Median PAV change: −3.7 vs. −1.5% (*p* = 0.006) Median TAV change: −9.3 vs. −1.7 mm^3^ (*p* < 0.001) Median lipid volume change: −3.4 vs. −1.3 mm^3^ (*p* = 0.284) Median calcification volume: −0.0 vs. 0 mm^3^ (*p* = 0.895)
Nicholls et al. [47] GLAGOV 2018	968 patients with SA	Evelocumab 420 mg (monthly) vs. placebo	76 weeks	Median PAV change: −1.2 vs. 0.6% (*p* < 0.001) Median TAV change: −3.6 vs. −0.8 mm^3^ (*p* = 0.04) Median necrotic core change: 0.13 vs. 0.46% (*p* = 0.67) Median dense calcium change: 2.2 vs. 1.4% (*p* = 0.10)
OCT				
Kataoka et al. [48] 2014	275 patients with SA	No statin vs. low statin vs. high statin (high statin therapy defined as atorvastatin >40 mg or rosuvastatin >20 mg)	Only baseline	Lipid arc: 238 vs. 219 vs. 161 (*p* = 0.03) Lipid length: 8.8 vs. 7.5 vs. 5.0 mm (*p* = 0.006) FCT: 74 vs. 91 vs. 116 μm (*p* < 0.01) TCFA: 52 vs. 20 vs. 8% (*p* < 0.001)
Komukai et al. [49] EASY-FIT 2014	70 patients with UA	20 mg vs. 5 mg atorvastatin	12 months	Median change in FCT: 69 vs. 17% (*p* < 0.001) Median change in lipid arc: −27% vs. −8% (*p* < 0.001) Decrease in macrophage grade: −38 vs. −24% (*p* < 0.001) Median lipid length change: −0.6 vs. −0.4 mm (*p* = NS)
Nishiguchi et al. [50] ESCORT 2017	70 ACS patients (53 included in final analysis)	4 mg pitavastatin from baseline vs. 4 mg pitavastatin 3 weeks after baseline	3 weeks and 36 weeks	Values given for OCT done after 3 weeks Median change in minimum FCT: 20 vs. −6 um (*p* < 0.05) Median change in maximum lipid arc: 5 vs. −5 (*p* = NS) Median change in lipid length: 0 vs. 0.6 mm (*p* = NS)
Raber et al. [51] IBIS-4 2019	103 patients with STEMI	Rosuvastatin 40 mg	13 months	Values compared to baseline Mean minimum cap thickness change: 21.41 um (*p* = 0.008) Mean cap thickness change: 69.26 um (*p* < 0.001) Mean macrophage lines arc change: −3.22 (*p* < 0.001) Mean lipid arc change: −12.49 (*p* = 0.013)
Kuroda et al. [52] 2019	48 patients with SA and ACS	Rosuvastatin 10 mg + 1800 mg EPA vs. rosuvastatin 2.5 mg	1 year	Median change in lipid length: −0.2 vs. 0.8 mm (*p* < 0.05) Median change in lipid arc: −2 vs. 19 (*p* < 0.05) Median change in lipid index: −45 vs. 217 (*p* < 0.05) Median change in macrophage grade: −16 vs. 18 (*p* < 0.05)
NIRS				
Kini et al. [53] YELLOW 2013	87 patients with SA	Intensive statin therapy (40 mg rosuvastatin) vs. standard of care	7 weeks	Median change in LCBI_4 mm_: −24.4 vs. 5.4% (*p* = 0.02)
Combined modalities				
Raber et al. [54] PACMAN-AMI 2022	300 patients with ACS	150 mg alirocumab (bi-weekly) vs. placebo	52 weeks	Median PAV change: −2.13 vs. −0.92% (*p* < 0.001) Median TAV change: −26.12 vs. −14.97 mm^3^ (*p* < 0.001) Median LCBI change: −29.3 vs. −12.38 (*p* = 0.004) Mean FCT change: 90.95 vs. 62.36 um (*p* = 0.03) Mean angular extension of macrophages change: −25.98 vs. –15.95 (*p* < 0.001)
Nicholls et al. [55] 2022	161 patients with NSTEMI (79 patients with IVUS analysis)	Evelocumab 420 mg (monthly) vs. placebo	52 weeks	Median minimum FCT change: 42.7 vs. 21.5 um (*p* = 0.015) Median maximum lipid arc change: −57.5 vs. −31.4 (*p* = 0.04) Median lipid length change: −5.8 vs. −3.3 mm (*p* = 0.02) Mean PAV change: −2.29 vs. −0.61 (*p* = 0.009) Mean TAV change: −19.0 vs. −8.9 mm^3^ (*p* = 0.04)

If not stated otherwise, values comparing treatment groups at follow-up. ACS, acute coronary syndrome; EPA, eicosapentaenoic acid; FCT, fibrous cap thickness; IVUS, intravascular ultrasound; LCBI, lipid core burden index; NC, necrotic core; NIRS, near-infrared spectroscopy; NSTEMI, non-ST segment elevation myocardial infarction; OCT, optical coherence tomography; PAV, percentage atheroma volume; SA, stable angina; STEMI, ST-segment elevation myocardial infarction; TAV, total atheroma volume; TCFA, thin cap fibroatheroma; UA, unstable angina; VH, virtual histology.
